# Novel lipoprotein density profiling in healthy dogs of various breeds, healthy miniature schnauzers, and miniature schnauzers with hyperlipidemia

**DOI:** 10.1186/1746-6148-9-47

**Published:** 2013-03-08

**Authors:** Panagiotis G Xenoulis, Paul J Cammarata, Rosemary L Walzem, Ronald D Macfarlane, Jan S Suchodolski, Jörg M Steiner

**Affiliations:** 1 Gastrointestinal Laboratory, Department of Small Animal Clinical Sciences, College of Veterinary Medicine and Biomedical Sciences, Texas A&M University, 4474 TAMU, College Station, TX 77843, USA; 2 Laboratory for Cardiovascular Chemistry, Department of Chemistry, Texas A&M University, 3255 TAMU, College Station 77842, USA; 3 Department of Nutrition and Food Science and Department of Poultry Science, Texas A&M University, 2253 TAMU, College Station, Texas 77843, USA

**Keywords:** Canine, Hypertriglyceridemia, Lipemia, Lipoprotein fingerprinting, NaBiEDTA, NaBiY, Ultracentrifugation, Disease

## Abstract

**Background:**

Despite the importance of abnormalities in lipoprotein metabolism in clinical canine medicine, the fact that most previously used methods for lipoprotein profiling are rather laborious and time-consuming has been a major obstacle to the wide clinical application and use of lipoprotein profiling in this species. The aim of the present study was to assess the feasibility of a continuous lipoprotein density profile (CLPDP) generated within a bismuth sodium ethylenediaminetetraacetic acid (NaBiEDTA) density gradient to characterize and compare the lipoprotein profiles of healthy dogs of various breeds, healthy Miniature Schnauzers, and Miniature Schnauzers with primary hypertriacylglycerolemia. A total of 35 healthy dogs of various breeds with serum triacylglycerol (TAG) and cholesterol concentrations within their respective reference intervals were selected for use as a reference population. Thirty-one Miniature Schnauzers with serum TAG and cholesterol concentrations within their respective reference intervals and 31 Miniature Schnauzers with hypertriacylglyceridemia were also included in the study.

**Results:**

The results suggest that CLPDP using NaBiEDTA provides unique diagnostic information in addition to measurements of serum TAG and cholesterol concentrations and that it is a useful screening method for dogs with suspected lipoprotein metabolism disorders. Using the detailed and continuous density distribution information provided by the CLPDP, important differences in lipoprotein profiles can be detected even among dogs that have serum TAG and cholesterol concentrations within the reference interval. Miniature Schnauzers with serum TAG and cholesterol concentrations within the reference interval had significantly different lipoprotein profiles than dogs of various other breeds. In addition, it was further established that specific lipoprotein fractions are associated with hypertriacylglyceridemia in Miniature Schnauzers.

**Conclusions:**

The results of the present study suggest that density gradient ultracentrifugation using NaBiEDTA is a useful screening method for the study of lipoprotein profiles in dogs. Therefore, this method could potentially be used for diagnostic purposes for the separation of dogs suspected of having lipoprotein abnormalities from healthy dogs.

## Background

The investigation of lipoprotein profiles in serum or plasma from healthy dogs has been the subject of occasional research since the 1940s [1-4]. Much of our current knowledge on canine lipoproteins originates from studies reported in the 1970s, which investigated dogs as possible models for human cardiovascular disease [3-5]. More recent studies investigated canine lipoproteins in association with several disease conditions or physiologic stages [5-11]. These studies have provided important information on the major serum lipoprotein fractions found in dog serum or plasma, namely chylomicrons, very low density lipoproteins (VLDL), low density lipoproteins (LDL), and high density lipoproteins (HDL).

The methodologies used to study canine lipoproteins in the past included electrophoresis, sequential density gradient centrifugation, and size exclusion methods [3-11]. These methods generally suffered from lack of resolution and could not capture detailed information related to the continuous density distribution of lipoproteins. In addition, highly specialized techniques of analytical ultracentrifugation and imaging were required to measure continuous density information. Technical and temporal barriers that limited research into the utility of continuous lipoprotein density profiles (CLPDP) in human and veterinary medicine have been largely overcome by recent developments in gradient-generating chemistries, centrifugation and imaging technologies [12,13]. At present, detailed and highly reproducible CLPDP are readily available [13]. The analytical resolution, speed and simplicity of these improved methods suggest that CLPDP may be suitable for rapid clinical and discovery purposes, particularly in settings where serum or plasma lipid concentrations alone are non-definitive. Such situations occur in both human [14,15] and animal [16,17] populations. In research settings there is high utility in the ability to rapidly screen profiles to identify interesting density subfractions for further compositional characterization [18,19]. Pragmatically, novel methods for lipoprotein analysis are slowly introduced to clinical practice for diagnostic purposes and used in clinical studies for risk assessment using combinations of analytical and statistical methods [18,19]. Such novel methods have not been previously applied to dogs, and therefore the feasibility of application and usefulness of these techniques is not known in this species.

Diseases that affect lipoprotein metabolism are both common and clinically important in dogs [20]. The most common disorders of lipoprotein metabolism in dogs are secondary to other diseases, such as diabetes mellitus, hypothyroidism, and hyperadrenocorticism [20]. Miniature Schnauzers are particularly interesting with regard to their serum lipids and lipoprotein profiles. Primary hypertriacylglycerolemia is a common condition in Miniature Schnauzers in the United States. In one study, hypertriacylglycerolemia was present in 32.8% of 192 Miniature Schnauzers investigated [21]. In this breed, hyperlipidemia, and more specifically hypertriacylgly-cerolemia, might be associated with diseases such as hepatobiliary disease, pancreatitis, insulin resistance, and ocular disease [16,17,20,22]. The biochemical, metabolic, and genetic bases of hypertriacylglycerolemia in Miniature Schnauzers have not been identified yet. Previous studies have shown that hypertriacylglycerolemia in Miniature Schnauzers is mainly characterized by an abnormal accumulation of VLDL with or without hyperchylomicronemia [10].

Despite the importance of abnormalities in lipoprotein metabolism in clinical canine medicine, the fact that most previously used methods for lipoprotein profiling are rather laborious and time-consuming has been a major obstacle to the wide clinical application and use of lipoprotein profiling in this species. Newer CLPDP techniques are not constrained by specific density intervals, require small volumes of serum or plasma, and can be completed in a few hours [12,13]. Coupled with rapid imaging of resolved lipoproteins and appropriate statistical analysis, these techniques are better able to capture diagnostic value from more fully resolved lipoprotein subclasses. To this end, a convenient, economical, and robust method of lipoprotein profiling that could be used for diagnostic purposes in clinical practice would be highly desirable. The aim of the present study was to assess the feasibility of a novel density gradient ultracentrifugation method in dogs, and to evaluate the ability of this method to separate healthy dogs of various breeds, healthy Miniature Schnauzers, and Miniature Schnauzers with primary hypertriacylglycerolemia based on their lipoprotein profiles.

## Methods

All owners participating in the study signed an informed owner consent form. The study protocol was reviewed and approved by the Clinical Research Review Committee at Texas A&M University (CRRC#08-37).

### Animals

#### Group 1: reference population composed of dogs of various breeds

A total of 35 healthy dogs of various breeds with serum triacylglycerol (TAG) and cholesterol concentrations within their respective reference intervals were selected for use as a reference population. Inclusion criteria included being a breed other than Miniature Schnauzer, being a breed that has not been reported to have any lipoprotein metabolism disorders [20], absence of any clinical signs at the time of blood collection and no history of disease or current use of drugs known to affect lipid metabolism. Serum TAG and cholesterol concentrations were measured in all dogs.

#### Group 2: miniature schnauzer dogs

Samples were selected from a pool of >300 samples from Miniature Schnauzers that were collected as part of several ongoing projects related to hypertriacylglyceridemia in this breed. All Miniature Schnauzers that were included in group 2 had to have absence of any clinical signs at the time of blood collection and no history of disease or use of drugs known to affect lipid metabolism. Serum TAG and cholesterol concentrations were measured in all dogs in group 2, and were used to categorize dogs into 2 subgroups.

##### Group 2A – normolipemic miniature schnauzers

Thirty-one Miniature Schnauzers with serum TAG and cholesterol concentrations within their respective reference intervals were included. These dogs were selected to be 7 years of age or older. An age criterion was imposed in order to minimize the possibility of them developing hyperlipidemia in the future because age is known to affect lipoprotein concentrations and distribution in this breed. Miniature Schnauzers that have not developed hypertriacylglyceridemia by the age of 7 years are unlikely to develop hypertriglyceridemia in the future [20].

##### Group 2B – hypertriacylglyceridemic miniature schnauzers

Thirty-one Miniature Schnauzers with serum TAG concentrations above the upper limit of the reference interval (>108 mg/dL) were also included in the study. In addition, in this group of dogs, serum canine specific pancreatic lipase (Spec cPL), glucose, total T4, and free T4 (in cases in which serum total T4 was below the lower limit of the reference interval) concentrations were measured to evaluate those dogs for any potential underlying diseases that may be responsible for hypertriacylglyceridemia. Based on the historical information for each dog and the results of the tests performed, all hypertriacylglyceridemic dogs enrolled in this study were diagnosed as having primary idiopathic hypertriacylglyceridemia of Miniature Schnauzers [20,21].

### Blood collection and handling

Owners living in relative proximity to the Gastrointestinal Laboratory at Texas A&M University were instructed to schedule an appointment for the blood collection at that location. Owners that could not come to Texas A&M for the blood collection were each sent a styrofoam box containing ice packs and the material necessary for blood collection, and were asked to schedule an appointment with their veterinarian for the blood collection. All owners were instructed not to feed their dogs for at least 12 hours before the scheduled blood collection. Ten milliliters of blood were collected from each dog into a red-top tube (with no additive). Immediately after clot formation, the samples were centrifuged and the serum was separated from the clot. Samples not collected at Texas A&M University were sent to the Gastrointestinal Laboratory packed on ice by overnight courier. Serum samples were stored at −80°C until analyzed.

### Questionnaires and consent forms

Owners of all dogs were asked to complete a questionnaire for each dog. Questions covered date of birth, sex, body weight, current diet(s), current medications, and current and past health history of the dogs. Questionnaires from all dogs were reviewed to determine whether the dogs fit the inclusion criteria for each group.

### Assays

Serum TAG (reference interval: 26–108 mg/dL), cholesterol (reference interval: 124–335 mg/dL), and glucose (reference interval: 60–120 mg/dL) concentrations were measured by automated enzymatic assays^a^. Serum Spec cPL concentrations (reference interval: ≤200 μg/L) were measured using an analytically validated immunoassay as described elsewhere [23]. Serum total T4 concentrations were measured by a solid-phase chemiluminescent competitive assay^b^. Serum free T4 concentration was measured using a commercial equilibrium dialysis radioimmunoassay^c^.

### Lipoprotein profile analysis

Lipoprotein profiling was carried out using a bismuth sodium ethylenediaminetetraacetic acid (NaBiEDTA) density gradient ultracentrifugation method as previously described with some modifications [24]. The sodium salt of BiEDTA has been described as a novel solute forming self-generating density gradient during ultracentrifugation of serum samples for the separation of lipoproteins [24]. Briefly, for each sample, 1,284 μL of a 0.18M NaBiEDTA^d^ gradient solution was added into a 1.5 mL tube. The fluorescent probe 6-((N-(7-nitrobenz-2-oxa-1,3-diazol-4-yl)amino)hexanoyl)sphingosine^e^ was reconstituted with 1 g/mL DMSO and ten μL of the 1 mg/mL solution were added to each tube to label the lipoproteins. Finally, 6 μL of serum was added to each tube to give a total volume of 1,300 μL. The mixture was vortexed at 1,400 rpm for 10 sec and 1,150 μL was transferred into an ultracentrifuge tube^f^ as this is the maximal amount of fluid retained during ultracentrifugation in the open top tube. The mixture was allowed to incubate for 30 minutes at 5°C to allow for the fluorescent probe to saturate the lipoproteins. The solution was then centrifuged at 120,000 rpm, at 5°C, for 6 hours in a Beckman Optima ultracentrifuge^g^ with a 30° fixed angle TLA 120.2 rotor^h^. A quality control sample was included in each run to verify proper operating conditions were achieved. Immediately after ultracentrifugation, the top of each sample was carefully layered with 250 mL of hexane to remove optical interference from the meniscus and imaged without delay.

For imaging, each tube was placed in a custom, in-house imaging instrument as previously described [24]. The samples were imaged using a custom-built fluorescence imaging system consisting of a digital camera^i^ with a MH-100 metal halide continuous light source^j^, located in a dark room. Two filters^k^ matching the excitation (blue-violet filter centered at 407 nm) and emission (a yellow emission long pass filter with a cut-on wavelength of 515 nm) characteristics of NBD C_6_-cermide were used. A gain of 1.0000, a target intensity of 30%, and an exposure time of 53.3 ms were selected. In order to be analyzed, the image of the each tube following ultracentrifugation was converted to a density profile using a commercially available software program^l^. A tube coordinate scale was established where 0.0 mm is the top of the tube and 34.0 mm is the bottom of the tube [24]. The average fluorescent intensity was then plotted as a function of tube coordinate.

### Statistical analysis

Commercial statistical software packages were used for all statistical analyses^m,n,o^. Data were analyzed for normal distribution using the Shapiro-Wilk test. Normally distributed data were reported as mean±SD and analyzed using t-tests. Not normally distributed data were reported as median and range and were analyzed using Mann–Whitney tests. Sliced inverse regression (SIR) was used to reduce the dimensionality of the density profile measurement and test the hypothesis that there is a relationship between group assignment and CLPDP [25]. Non-parametric correlations were used to test a linear relationship between parameters. Significance was set at p<0.05 for all analyses.

## Results

### Signalment of dogs

The breed, sex, sexual status, body condition score (BCS), and age of the enrolled dogs are shown in Table 1.

**Table 1:** Signalment of dogs

**Parameter**	**Reference population, Group 1**	**Miniature Schnauzers**	
		**Group 2A**	**Group 2B**
Pure Breed, n (number of breeds)	23 (19)	31 (1)	31 (1)
Mixed Breed, n	12	0	0
Males, n (castrated)	19 (16)	16 (5)	12 (12)
Females, n (spayed)	16 (15)	15 (9)	19 (16)
BCS*, Median (range)	5 (3.5 - 6.0)	5 (3.9 – 7.0)	5 (4.9 – 7.9)
Age**, Median (range)	4.4 (1.3 - 11.9)	9.3 (7 – 12)	9.5 (4.9 – 9.0)

*BCS = Body Condition Score; Values can range from 1 to 9; 5 is considered ideal and 9 is considered obese.

** Age is reported in years.

There were no statistically significant differences in BCS among the three groups (p=0.478). Dogs in group 1 were significantly younger than dogs of group 2A (p<0.0001) and group 2B (p<0.0001). There was no statistically significant difference in age between groups 2A and 2B (p=1.0). Also, there were no statistically significant differences in the dogs’ sex between any of the groups (all p-values >0.05).

### Serum TAG and cholesterol concentrations

**Figure 1:**
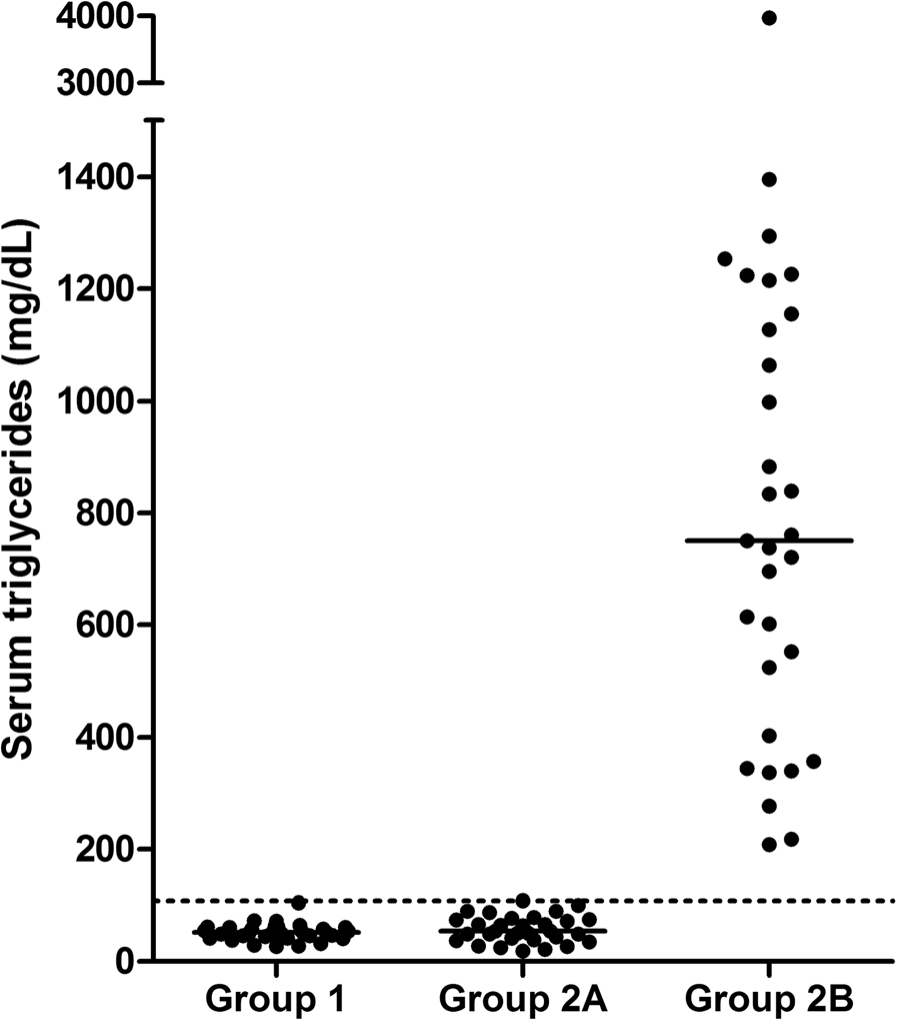
**Serum TAG concentrations in dogs of groups 1, 2A, and 2B.** Serum TAG concentrations of dogs in groups 1 and 2A were all within the reference interval. Serum TAG concentrations of dogs in group 2B were all above the upper limit of the reference interval. The dashed line represents the upper limit of the reference interval and the solid lines represent the median for each group.

**Figure 2:**
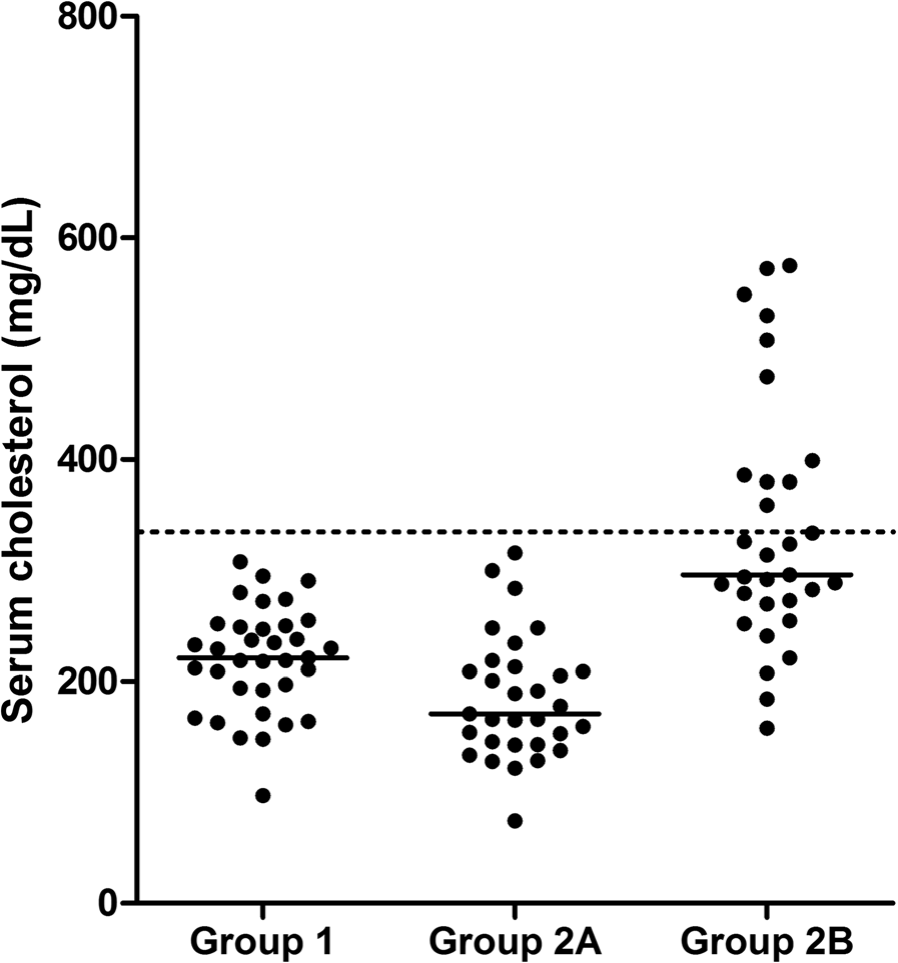
**Serum cholesterol concentrations in dogs of groups 1, 2A, and 2B.** Serum cholesterol concentrations of dogs in groups 1 and 2A were all within the reference interval. Serum cholesterol concentrations were above the upper limit of the reference interval for 11 dogs in group 2B. The dashed line represents the upper limit of the reference interval and the solid lines represent the median for each group.

Serum TAG concentrations of dogs in group 1 (median: 52 mg/dL; range: 27–105 mg/dL), were not significantly different from those of dogs in group 2A (median: 54 mg/dL; range: 19–108 mg/dL; p value > 0.05; Figure 1). Serum cholesterol concentrations of dogs in group 1 (median: 221 mg/dL; range: 97–308 mg/dL) were significantly higher than those of dogs in group 2A (median: 168 mg/dL; range: 74–316 mg/dL; p value= 0.003; Figure 2), although all values for both groups were within the reference interval.

Serum TAG concentrations of dogs in group 2B ranged from 218 mg/dL to 3,975 mg/dL (median: 750 mg/dL). Eleven of the 31 dogs (35%) also had hypercholesterolemia. Serum cholesterol concentrations ranged from 158 mg/dL to 575 mg/dL (median: 296 mg/dL). As expected, both serum TAG (p<0.05) and cholesterol (p<0.05) concentrations were significantly higher in dogs of group 2B than dogs of group 2A (Figures 1 and 2). Similarly, both serum TAG (p<0.05) and cholesterol (p<0.05) concentrations were significantly higher in dogs of group 2B than dogs of group 1 (Figures 1 and 2).

### Continuous lipoprotein density profiling

Eleven separate lipoprotein fractions were identified solely on density characteristics and not on their functional properties or composition. In fact, for most of those lipoprotein fractions, the functional characteristics and composition are currently unknown. Therefore, all assignments within the lipoprotein fingerprint to traditional functional classes, e.g. LDL or HDL, were strictly considered nominal. Density ranges were classified as R1 to R11 as follows: R1 (*d*<1.017 g/mL), R2 (*d*=1.019 to 1.023 g/mL), R3 (*d*=1.023 to 1.029 g/mL), R4 (*d*=1.029 to 1.039 g/mL), R5 (*d*=1.039 to 1.050 g/mL), R6 (*d*=1.050 to 1.063 g/mL), R7 (*d*=1.063 to 1.091 g/mL), R8 (*d*=1.091 to 1.110 g/mL), R9 (*d*=1.110 to 1.133 g/mL), R10 (*d*=1.133 to 1.156 g/mL), and R11 (*d*=1.156 to 1.179 g/mL) [26]. Based on a previously published classification and based solely on their density characteristics, these fractions could be classified as: TAG-reach lipoproteins (TRL; chylomicrons and VLDL; *d*<1.017 g/mL), LDL_1_ (*d*=1.019 to 1.023 g/mL), LDL_2_ (*d*=1.023 to 1.029 g/mL), LDL_3_ (*d*=1.029 to 1.039 g/mL), LDL_4_ (*d*=1.039 to 1.050 g/mL), LDL_5_ (*d*=1.050 to 1.063 g/mL), HDL_2b_ (*d*=1.063 to 1.091 g/mL), HDL_2a_ (*d*=1.091 to 1.110 g/mL), HDL_3a_ (*d*=1.110 to 1.133 g/mL), HDL_3b_ (*d*=1.133 to 1.156 g/mL), and HDL_3c_ (*d*=1.156 to 1.179 g/mL), respectively [26]. HDL_1_ is typically not found in healthy humans but it does occur in healthy dogs [4,26]. However, it has not been convincingly shown that the previously described canine HDL_1_ molecule has the same function as human HDL_1_, and the density range of canine HDL_1_ has not been accurately determined (previously published densities vary between 1.025 and 1.1) [4,27]. Determination of the density interval of that fraction by additional compositional analysis was not an aim of the present study.

#### Continuous lipoprotein density profiles of groups 1 and 2A dogs

**Figure 3:**
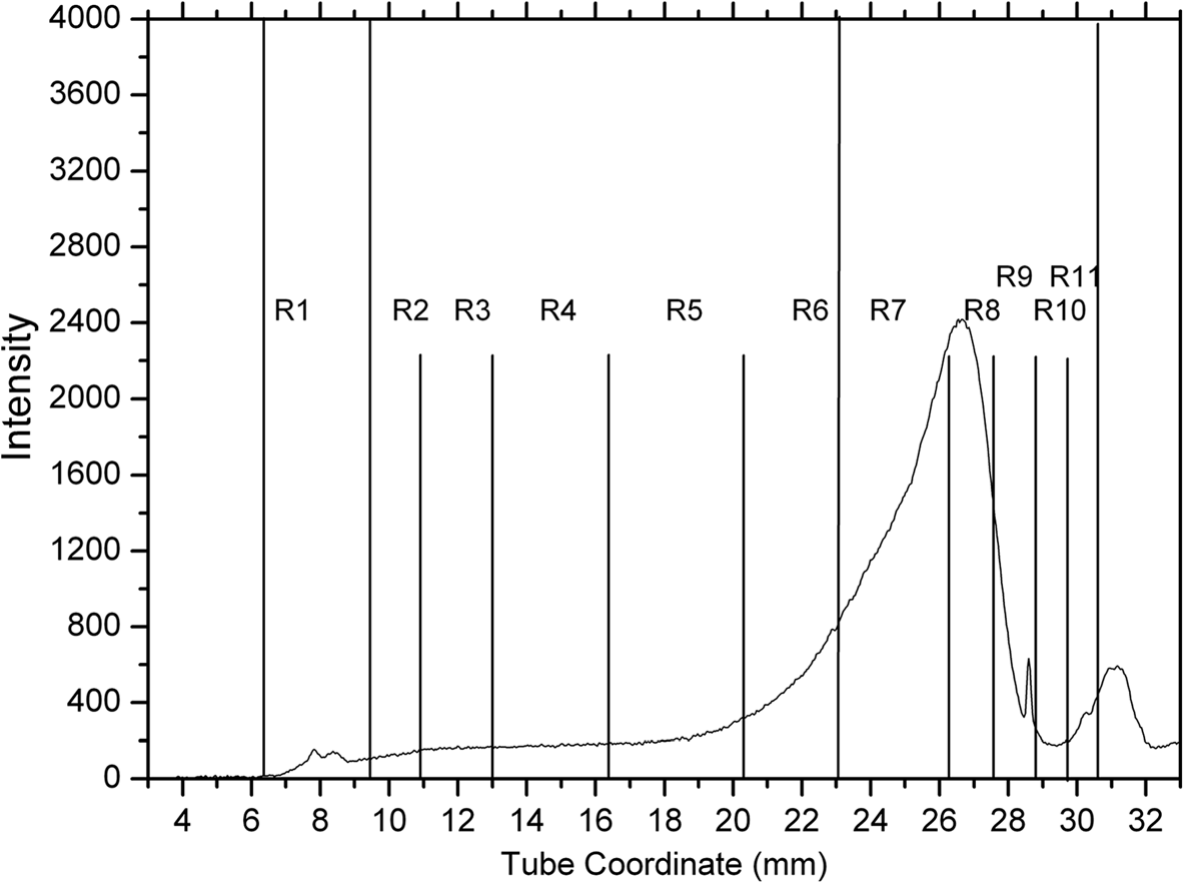
**Representative continuous lipoprotein density profile from a dog of group 1 (normolipemic dogs of breeds other than Miniature Schnauzers).** Intensity refers to the number of pixels of fluorescence emission recorded. The x-axis provides the distance in mm from the top of the 33 mm ultracentrifuge tube. This dog had serum TAG and cholesterol concentrations within the respective reference intervals. The most abundant lipoprotein fractions are R7 and R8 (which correspond to a density for HDL), seen at a tube coordinate between 23 mm and 31 mm. R2 to R6 fractions (corresponding to LDL densities) are present in very small amounts and are seen at a tube coordinate between 9 mm and 23 mm. The R1 fraction (that corresponds to TRL densities) appears at tube coordinates of 6 mm to 9 mm in very small amounts.

Figure 3 shows a representative lipoprotein profile from a dog in group 1. It is clearly evident that the most abundant lipoprotein fractions are R7 to R11 (d=1.063-1.0179), seen at a tube coordinates between 23 mm and 31 mm, and most likely represent HDL. R2 to R6 fractions, most likely representing LDL, were seen at a tube coordinates between 9 mm and 23 mm, but they were present in very small amounts, with the exception of R5 and R6 (d = 1.038-1.063 g/mL). The R1 fraction, most likely representing TRL (d ≤ 1.017 g/mL) was seen at a tube coordinates of 6 mm to 9 mm and was presumed to contain chylomicrons, VLDL, and chylomicron and VLDL remnants. The TRL fraction was present in very small amounts.

**Figure 4:**
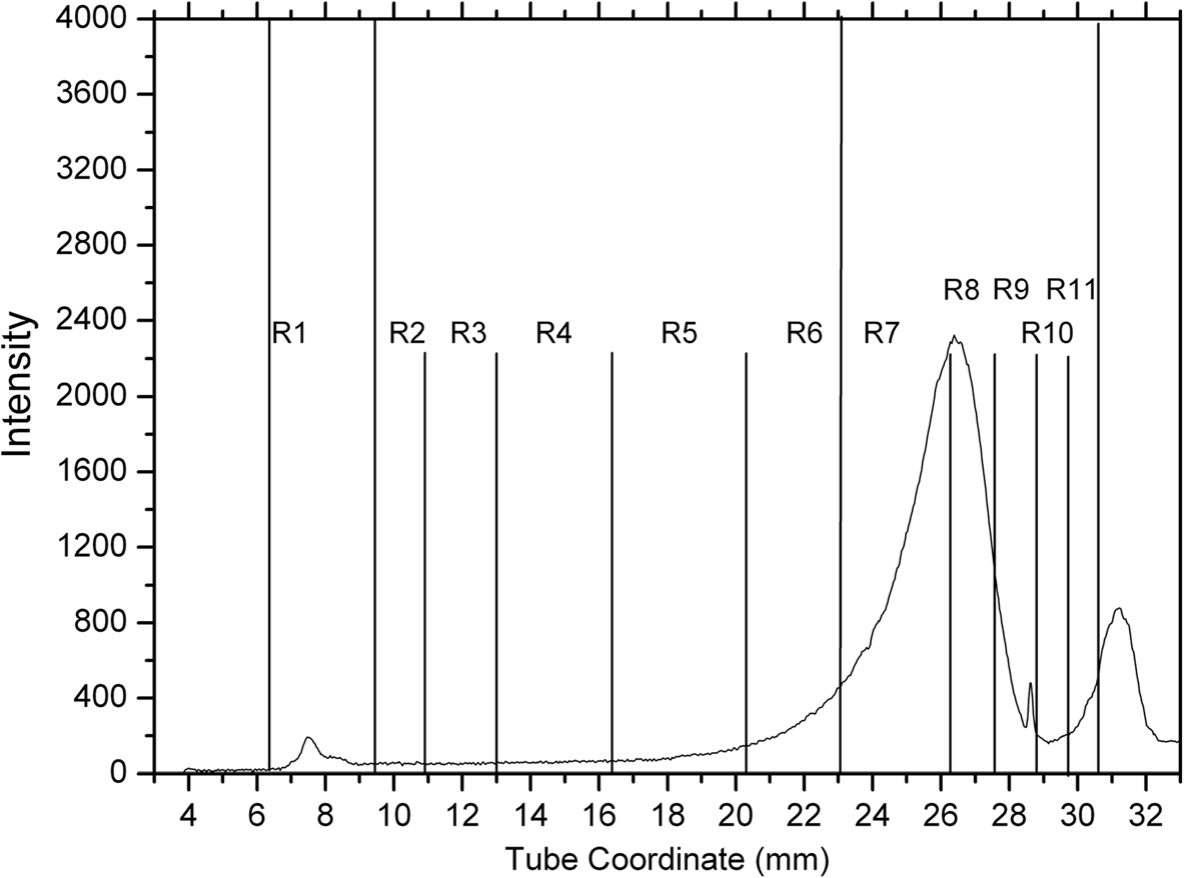
**Representative continuous lipoprotein density profile from a dog of group 2A (normolipemic Miniature Schnauzers).** Axes descriptions are provided in Figure 4 legend. This Miniature Schnauzer had serum TAG and cholesterol concentrations within the respective reference intervals. The CLPDP of these dogs were generally similar to those of group 1 (Figure 4) with regard to the abundance of major lipoprotein classes. However, most dogs in group 2A showed more abundant R1 and less abundant R2 – R6 fractions compared to group 1 dogs.

Figure 4 shows a representative CLPDP from a dog in group 2A. The lipoprotein profiles of Miniature Schnauzers with serum TAG and cholesterol concentrations within the reference interval (group 2A) were generally similar to the ones seen in dogs of group 1 with regard to the abundance of major lipoprotein classes. However, most dogs in group 2A showed some distinct differences in some lipoprotein fractions that were further analyzed by SIR.

**Figure 5:**
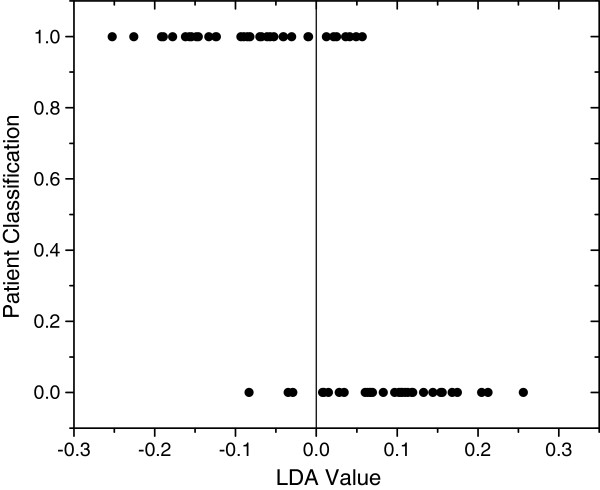
**One dimensional SIR plot showing classification of dogs into groups based on CLPDP.** The vertical line separates the two groups based on the measured multidimensional characteristic (i.e., CLPDP). A linear discriminant analysis (LDA) value generated for each dog ranks the individual within the group relative to the vertical line. Individual dogs are represented as dots. The dogs represented by the dots that are at the bottom of the graph are the Miniature Schnauzers of group 2A. Their lipoprotein profiles plot them all to the right of the vertical line, with the exception of 3 dogs (90% of dogs classified correctly). The dogs represented by the dots at the top of the graph are the dogs of other breeds (group 1). Twenty-eight of the 35 dogs (80%) were classified as a separate group to the left of the vertical line. Seven of the 35 dogs were classified to the right of the vertical line together with the Miniature Schnauzers.

Sliced inverse regression analysis was used to predict if differences in lipoprotein profiles were present between groups 1 and 2A, and also to test whether lipoprotein profiles were effective in predicting which group each dog belonged to. Based on the classification table that documents the validity of predicted probabilities, the group to which each dog belonged (i.e., Miniature Schnauzer versus other breed) could be accurately predicted based on their lipoprotein profiles in 85% of the cases (Eigenvalues=0.5455; p=0.00017; Figure 5). Specifically, 90% of Miniature Schnauzers could be classified as Miniature Schnauzers, and 80% of dogs of other breeds could be classified as other breeds based on their lipoprotein profiles alone. The most important lipoprotein fractions that served as predictors were the R1, and R5 - R6 (d = 1.038-1.063 g/mL) (Figures 3 and 4). Normolipemic Miniature Schnauzers had more prominent R1 (likely TRL) peaks than dogs of other breeds, while dogs of other breeds had more prominent R5 and R6 peaks (likely nominal LDL_4_ and LDL_5_ peaks).

#### Continuous lipoprotein density profiles of group 2B dogs

**Figure 6:**
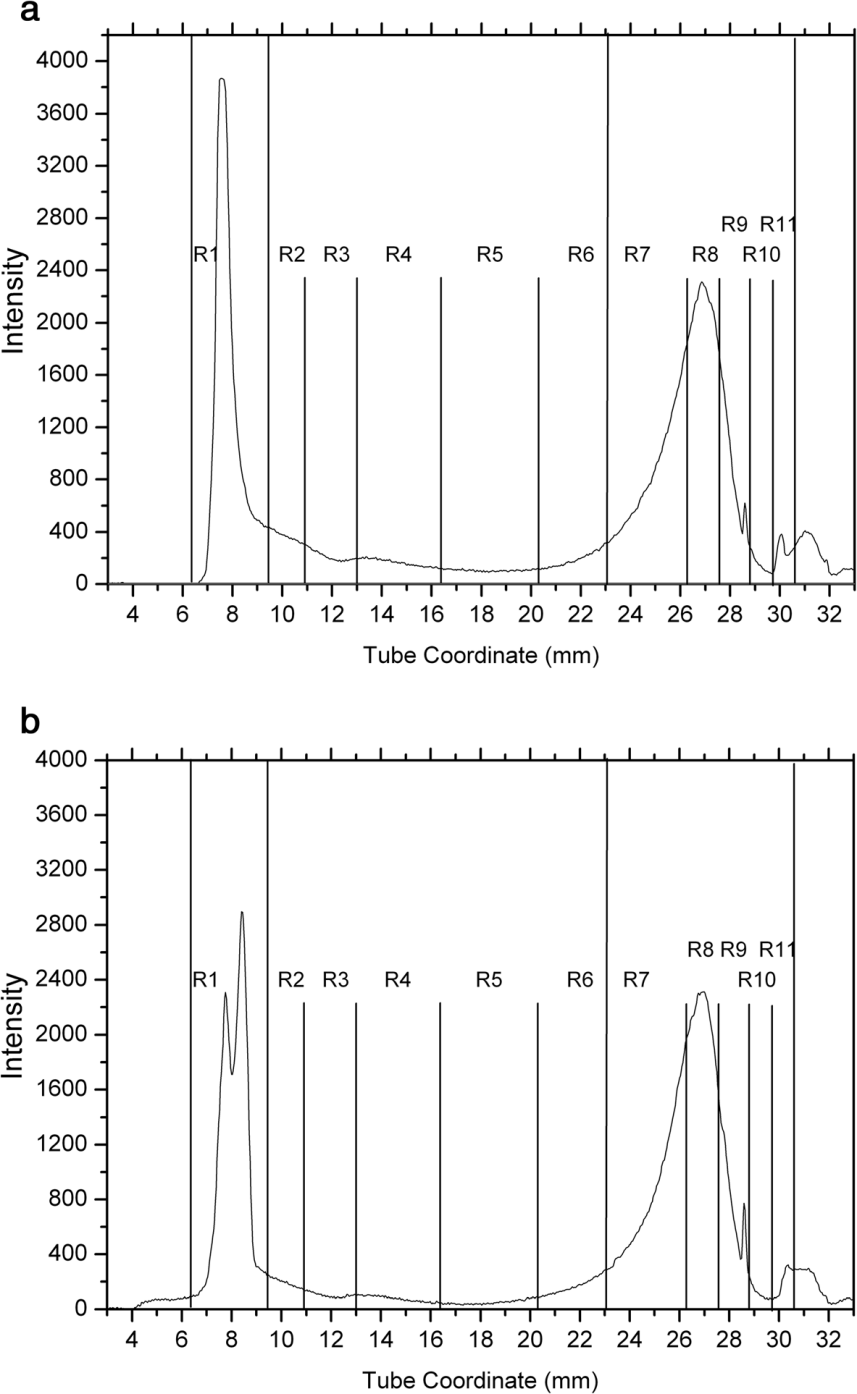
**Representative continuous lipoprotein density profiles from 2 dogs (a and b) of group 2B (hyperlipidemic Miniature Schnauzers).** As in dogs in groups 1 and 2A, fractions corresponding to nominal HDL densities (R7 to R11) were abundant while fractions corresponding to nominal LDL densities (R2 to R6) were low in this group. However, dogs of this group had prominent R1 peaks corresponding to the TRL area. Note the difference in the peak shapes of R1 fractions between the two dogs (**a** versus **b**).

Figures 6a and 6b show representative lipoprotein profiles from two dogs in group 2B. Similarly to dogs in groups 1 and 2A, R7 – R11 fractions (likely corresponding to HDLs) were abundant and R2 – R6 fractions (likely corresponding to LDLs) were low in this group. However, dogs of this group had prominent R1 peaks likely corresponding to the TRL area.

**Figure 7:**
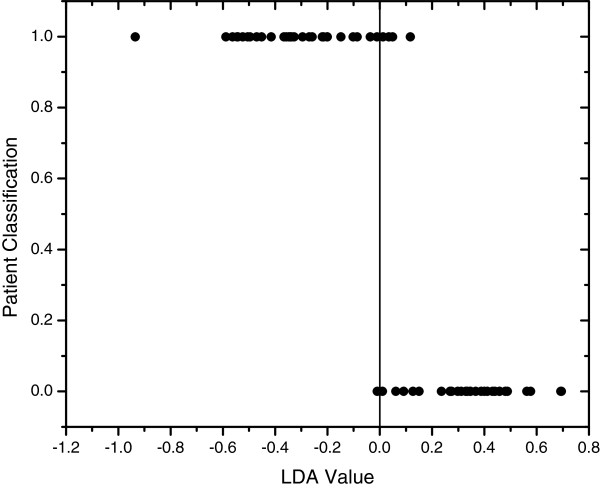
**One dimensional SIR plot showing classification of dogs into groups based on CLPDP.** The vertical line separates the two groups based on the measured multidimensional characteristic (i.e., CLPDP). A linear discriminant analysis (LDA) value generated for each dog ranks the individual within the group relative to the vertical line. Individual dogs are represented as dots. Group 2B hyperlipidemic Miniature Schnauzers are represented by the dots that are at the bottom of the graph. Their CLPDPs plot them all to the right of the vertical line, with the exception of one dog with mildly increased serum TAG concentration (97% of dogs classified correctly). Group 2A normolipemic Miniature Schnauzers are represented by the dots at the top of the graph are the Miniature Schnauzers. All but 2 of the Group 2A dogs are classified as a separate group to the left of the vertical line (94% of dogs classified correctly).

Sliced inverse regression analysis was used to predict if differences in lipoprotein profiles were present between groups 2A and 2B, and also to test whether and which lipoprotein profiles were effective in predicting which group each dog belonged to. The SIR model showed that the group to which each dog belonged (i.e., Miniature Schnauzers with normal versus hypertriacylglyceridemic) could be accurately predicted based on their lipoprotein profiles in 95% of cases (Eigenvalues=0.7638; p=0.000002; Figure 7). Specifically, 97% of non- hypertriacylglyceridemic Miniature Schnauzers were correctly classified, and 94% of hypertriacylglyceridemic Miniature Schnauzers were correctly classifiedbased on their CLPDP data alone. By far, the most important lipoprotein fraction that served as a predictor was the TRL fraction, which was more prominent in the dogs with hypertriacylglyceridemia. Fractions corresponding to R3, R4, and R5 (1.023-1.050 g/mL) were more prominent in Miniature Schnauzers with serum TAG concentrations within the reference interval (Figures 6 and 7).

### Correlations

Non-parametric correlation tests were used to test whether there was a linear relationship between lipoprotein fractions that showed significance in the SIR models and serum TAG and/or cholesterol concentrations. There was a significant positive correlation between the nominal TRL (R1) intensity with both serum TAG concentration (Spearman r=0.81; 95% CI=0.73-0.87; p<0.0001) and serum cholesterol concentration (Spearman r=0.61; 95% CI=0.46-0.72; p<0.0001). There were also significant, but weak, positive correlations between serum cholesterol concentration and the nominal LDL_2_ (R3) fraction (Spearman r=0.42; 95% CI=0.24-0.58; p<0.0001) and LDL_4_ (R5) (Spearman r=0.31; 95% CI=0.11-0.48; p=0.0023).

## Discussion

The method presented here was easy to perform and proved to be a quick and accurate screening method for lipoprotein analysis in dogs. Important differences in lipoprotein profiles between different groups of dogs were detected with this method. An important and novel finding of the present study is that Miniature Schnauzers with serum TAG and cholesterol concentrations within the reference interval have significantly different lipoprotein profiles than those of dogs of various other breeds. In addition, the present study confirmed and expanded the findings of previous studies reporting that specific lipoprotein classes are associated with hypertriacylglyceridemia in Miniature Schnauzers.

To our knowledge, this is the first study showing that Miniature Schnauzers with normal serum TAG and cholesterol concentrations differ significantly in certain lipoprotein fractions (R1 and R5 possibly corresponding to TRL and LDL_4_) from dogs of various other breeds. A previous study [10] identified a small number of healthy Miniature Schnauzers (4 out of 11 studied) that differed in some lipoprotein fractions (LDL and VLDL) compared with dogs of other breeds. However, in that particular study, Miniature Schnauzers were classified as non-lipemic based on the gross appearance of the plasma rather than a measured TAG concentration. Indeed, all 4 of the non-lipemic Miniature Schnauzers classified as “different” had mild increases in plasma TAG concentrations, and therefore, mild lipid metabolism alterations were present.

The R1 fraction (likely TRLs) was significantly higher in Miniature Schnauzers of group 2A than in dogs of other breeds, despite the fact that there was no significant difference in serum TAG concentrations between the two groups and serum TAG and cholesterol concentrations were within the reference interval. Interestingly, serum cholesterol concentrations were found to be significantly higher in dogs of other breeds compared to Miniature Schnauzers. This difference might be related to the fact that the R5 fraction was significantly higher in dogs of other breeds compared to Miniature Schnauzers. However, similarly to serum TAG concentrations, serum cholesterol concentrations were all within the reference interval. The density interval of R5 was d = 1.039-1.050 g/mL, a density region that can include LDL_4_ but also large buoyant HDL [28]. The marked differences in lipoprotein profiles between Miniature Schnauzers and dogs of other breeds despite the normal serum TAG and cholesterol concentrations clearly suggest that serum TAG and cholesterol concentrations are insensitive markers for detecting differences in lipoprotein metabolism in dogs. These differences in lipoprotein profiles could be identified in the vast majority of dogs, as about 90% of them could be classified to the correct group based on their lipoprotein profile alone.

The reason for the differences in lipoprotein profiles between Miniature Schnauzers and dogs of other breeds with normal serum TAG and cholesterol concentrations is unknown. The clinical importance of such finding is also unknown. One plausible scenario for such difference is that some Miniature Schnauzers might have an early disorder in lipoprotein metabolism that has not yet affected the overall serum TAG and cholesterol concentrations, but these concentrations might be affected in the future. This hypothesis is supported by the findings of a previous study that suggested that hyperlipidemia is an age-related condition in Miniature Schnauzers [21]. However, this does not seem a likely explanation in this study because all Miniature Schnauzers with serum TAG concentrations within the reference interval enrolled into this study were above 7 years of age (median age: 9.3 years). Therefore, these dogs were already of a rather advanced age and unlikely to develop hypertriacylglyceridemia later in life [21]. Another possibility is that the majority of Miniature Schnauzers differ in their basic lipoprotein metabolism from dogs of other breeds but only a portion of these dogs have severe enough lipid metabolism disorders leading to hyperlipidemia. Also, differences in diet composition or may have accounted, at least in part, for the differences in lipoprotein profiles. Differences in the sexual status of the dogs between groups may also have played a role although this has not been demonstrated in previous studies. Clearly, further studies are needed to determine the reason for this finding and its clinical significance. Moreover, the ability to rapidly screen the entire density profile of serum lipoproteins has identified specific density regions amenable to further compositional studies that may identify the mechanism for the distinctive lipoprotein metabolism that occurs in Miniature Schnauzers.

Lipoprotein profiles of Miniature Schnauzers with hypertriacylglyceridemia were in agreement with the findings of previous studies [10]. It is interesting to note that the main difference between hyperlipidemic and normolipidemic Miniature Schnauzers was a significant increase of the R1 (TRL) fraction in hyperlipidemic Miniature Schnauzers, and there was a strong correlation between TRLs and serum TAG concentrations. Similarly to previous studies, there was no difference in the HDL fractions [10]. Interestingly, the present study showed that a specific fraction (R3 likely corresponding to the density of LDL_2_) was significantly decreased in Miniature Schnauzers with hyperlipidemia.

Another interesting observation is that the CLPDP of Miniature Schnauzers with hyperlipidemia, although different in their lipoprotein profiles from Miniature Schnauzers without hyperlipidemia, was rather diverse and there were some distinct differences among dogs in the same group. These differences were not always related to the different degrees of hyperlipidemia in these dogs. For example, many hyperlipidemic Miniature Schnauzers had 2 distinct peaks in their TRL fractions (Figure 7), while others only had one (Figure 6). These 2 peaks likely represent chylomicrons and VLDLs, which have slightly different densities. This is in agreement with findings of an older study [10], in which it was shown that some hyperlipidemic Miniature Schnauzers had increases in VLDLs only, while others had increases in both VLDLs and chylomicrons. It is not known why some of the hyperlipidemic Miniature Schnauzers have only one TRL fraction affected while others have 2. In addition, as shown in previous studies [10,21], a fraction if these dogs had increases in serum cholesterol concentrations. Thus, it is obvious that hyperlipidemia in Miniature Schnauzers is not a phenotypically uniform disease. This might be the result of the effect of environmental factors or maybe due to genetic heterogeneity.

Miniature Schnauzers of either group were significantly older than the dogs of other breeds used in this study. This was the result of specific selection criteria. Hyperlipidemia in Miniature Schnauzers is known to be an age-related condition and, therefore, dogs in group 2 had to be of older age in order to have developed their phenotype with a high degree of probability if in fact they were to develop hypertriacylglyceridemia. For example, in one study, only 16% of Miniature Schnauzers between 1 and 3 years of age were hypertriacylcerolemic, while 78% of Miniature Schnauzers >9 years of age were hypertriacylglycerolemic. No association between age and serum TAG or cholesterol concentrations have been reported or suspected in dogs of breeds other than Miniature Schnauzers.

It needs to be pointed out that the present method as described in the present study does not allow detailed characterization of the components of each lipoprotein fraction. As mentioned above, the lipoprotein fractions were identified solely based on density characteristics and not on their functional properties or composition, which are to a large degree unknown in dogs. Therefore, all assignments within the lipoprotein fingerprint to traditional functional classes, e.g. LDL or HDL, were strictly considered nominal. Further studies are warranted to investigate the exact content and functional properties of all canine lipoprotein fractions.

## Conclusions

In conclusion, the results of the present study suggest that density gradient ultracentrifugation using NaBiEDTA is a useful screening method for the study of lipoprotein profiles in dogs. Therefore, this method could potentially be used for diagnostic purposes for the separation of dogs suspected of having lipoprotein abnormalities from healthy dogs. Important differences in lipoprotein profiles can be detected with this method even among dogs that have serum TAG and cholesterol concentrations within the reference interval, and dogs belonging to different groups can be effectively separated based on their lipoprotein profiles using discriminant analysis. Miniature Schnauzers with serum TAG and cholesterol concentrations within the reference interval had significantly different lipoprotein profiles (mainly with regard to fractions R1 and R5, which based on density characteristics, correspond to TRL and LDL_4_) than dogs of various other breeds. In addition, it was further established that specific lipoprotein fractions (R1 and R3, which based on density characteristics, correspond to TRL and LDL_2_) are associated with hypertriacylglyceridemia in Miniature Schnauzers. Changes in these lipoprotein fractions are not always uniform among Miniature Schnauzers with hyperlipidemia. Further studies are needed to evaluate the usefulness of density gradient ultracentrifugation using NaBiEDTA in evaluating hyperlipidemia of other causes in dogs, and to establish the clinical significance of differences in lipoprotein profiles in Miniature Schnauzers.

## Endnotes

^a^Roche/Hitachi MODULAR ANALYTICS D 2400 module, Roche Diagnostics, Indianapolis, IN; ^b^Immulite 2000 Canine Total T4, Siemens Healthcare Diagnostics, Deerfield, IL; ^c^Free T4 (by ED), Antech Diagnostics, Irvine, CA; ^d^Bismuth Sodium Ethylenediaminetetraacetate, TCI AMERICA, Portland, OR; ^e^NBD C_6_-ceramide; NBD C_6_-ceramide, Molecular Probes, Inc. Eugene, OR; ^f^Thickwall, Polycarbonate (1 mL, 11 x 34 mm), Beckman Coulter Inc., Brea, CA; ^g^TLX-110; Beckman Coulter Optima TLX-120 Ultracentrifuge, Beckman Coulter Inc., Brea, CA; ^h^Beckman Coulter Inc, Brea, CA; ^i^Digital Microfire Camera, Optronics, Goleta, CA; ^j^MH-100, Dolan-Jenner Industries, Boxborough, MA; ^k^SCHOTT North America, Inc., Elmsford, NY; ^l^Origin 7.0, Microcal Software Inc., Northampton, MA; ^m^SPSS 16.0, SPSS Inc., Chicago, IL; ^n^Prism5, GraphPad, San Diego, CA; ^o^R, http://www.r-project.org/.

## Competing interests

The authors declare that they have no competing interests.

## Authors’ contributions

PGX conceived, designed, and conducted the study, analyzed the data, and wrote the paper. PC contributed to acquisition of data, analyzed the data, and revised the paper. RLW, RM, JSS, and JMS contributed to acquisition of data and revised the paper. All authors read and approved the final manuscript.
